# Altered Relationship Between Parvalbumin and Perineuronal Nets in an Autism Model

**DOI:** 10.3389/fnmol.2021.597812

**Published:** 2021-04-12

**Authors:** Dan Xia, Li Li, Binrang Yang, Qiang Zhou

**Affiliations:** ^1^Key Laboratory of Chemical Genome, State Key Laboratory of Chemical Oncogenomics, School of Chemical Biology and Biotechnology, Shenzhen Graduate School, Peking University, Shenzhen, China; ^2^Center for Child Care and Mental Health, Shenzhen Children’s Hospital, Shenzhen, China; ^3^State Key Laboratory of Organ Failure Research, Department of Biostatistics, Guangdong Provincial Key Laboratory of Tropical Disease Research, School of Public Health, Southern Medical University, Guangzhou, China

**Keywords:** parvalbumin, perineuronal net, autism spectrum disorder, valproic acid, chondroitinase ABC

## Abstract

Altered function or presence of inhibitory neurons is documented in autism spectrum disorders (ASD), but the mechanism underlying this alternation is poorly understood. One major subtype of inhibitory neurons altered is the parvalbumin (PV)-containing neurons with reduced density and intensity in ASD patients and model mice. A subpopulation of PV^+^ neurons expresses perineuronal nets (PNN). To better understand whether the relationship between PV and PNN is altered in ASD, we measured quantitatively the intensities of PV and PNN in single PV^+^ neurons in the prelimbic prefrontal cortex (PrL-PFC) of a valproic acid (VPA) model of ASD at different ages. We found a decreased PV intensity but increased PNN intensity in VPA mice. The relationship between PV and PNN intensities is altered in VPA mice, likely due to an “abnormal” subpopulation of neurons with an altered PV-PNN relationship. Furthermore, reducing PNN level using *in vivo* injection of chondroitinase ABC corrects the PV expression in adult VPA mice. We suggest that the interaction between PV and PNN is disrupted in PV^+^ neurons in VPA mice which may contribute to the pathology in ASD.

## Introduction

Autism Spectrum Disorders (ASD) are a group of neurodevelopmental disorders that share core clinical symptoms in social communication deficits and repetitive behaviors (Lord et al., 2018). The prevalence of the disease has been increasing, from 1% reported in 2012 to 1.5% in developed countries (Lyall et al., 2017). However, the etiology of ASD is poorly understood. ASD is viewed as a multifactorial disease caused by a combination of environmental and genetic cues. Environmental insults during embryonic development and early postnatal life are believed to play an important role in ASD pathophysiology. *In-utero* exposure of rodents to valproic acid (VPA) represents a robust model of autism exhibiting behavioral phenotypes in juvenile VPA rats and mice and persist into adulthood, all related to core human ASD symptoms (Ornoy et al., 2016). This model might better mimic idiopathic ASD. In the developing brain, alterations in synapse formation, synaptic function, and dendritic growth result in neuronal network dysfunction, leading ultimately to social and cognitive dysfunctions. Dysregulation of synaptic plasticity and neuronal activity was found in ASD (Gilbert and Man, 2017). In the VPA rat model, a reduction in dendritic spine density in the prefrontal cortex (PFC) but an increase in spine number in the ventral hippocampus was reported (Bringas et al., 2013; Nicolini and Fahnestock, 2018).

Inhibitory GABAergic neurons expressing parvalbumin (PV^+^ neurons) and associated perineuronal nets (PNN) are important regulators of experience-dependent plasticity throughout life (Caroni et al., 2012). PV^+^ neurons in the cortex are essential players in generating gamma oscillations which are believed to participate in many critical functions such as working memory (Buzsáki and Wang, 2012). Selectively reducing PV^+^ neuronal activity strongly attenuates gamma-band oscillations, a phenomenon often observed in ASD patients during cognitive tasks (Sohal et al., 2009). Also, PV-deficient (PV^+/–^ and PV^−/−^) mice showed a striking ASD phenotype (Wöhr et al., 2015). PNNs are condensed extracellular matrix structures preferentially wrapping around the soma and proximal dendrites of PV^+^ neurons (Reichelt et al., 2019). The intensity of PNN [labeled by *Wisteria floribunda* agglutinin (WFA)] is commonly used as an indirect measure of their developmental maturity, with bright staining representing a mature PNN while dim staining representing an immature PNN (Wang and Fawcett, 2012). Most studies on PNN have revealed their contributions to neural plasticity (e.g., critical period plasticity; Pizzorusso et al., 2002; Levelt and Hübener, 2012), memory (Banerjee et al., 2017), brain diseases (such as schizophrenia, drug addiction, and neurodegenerative diseases; Mauney et al., 2013; Pantazopoulos and Berretta, 2016), and protect neurons against oxidative stress (Cabungcal et al., 2013). The maturation of PV^+^ neurons is accompanied by the condensation of PNN, which forms a mesh-like structure perforated by synapses, surrounding the somata and proximal neurites of these cells (Miyata et al., 2012; Favuzzi et al., 2017). The condensed PNN is thought to act as a molecular brake on synaptic plasticity as critical periods close (Wang and Fawcett, 2012; Reichelt et al., 2019), and cleavage of PNN components can partially restore plasticity in adulthood (Pizzorusso et al., 2002) or resulted in an enhanced but dysregulated plasticity (Cisneros-Franco et al., 2018). Reduced density of PV^+^ neurons was found in the postmortem PFC tissue from 11 autistic cases (Hashemi et al., 2017) and the PNN was not detected yet. The abnormalities of PNN and PV in ASD may vary between models: Shank3B^−/−^ ASD model mice (Filice et al., 2016) and VPA model mice (Lauber et al., 2016) showed reduced expression of PV mRNA and protein without alterations in the densities of PV^+^ neurons and PNN (*Vicia Villosa* Agglutinin-positive) at P25; *MeCP2*^−/−^ ASD model mice showed an increased density of PNN (WFA-positive) in visual cortex at P15 and P30 and accelerated maturation of PV^+^ neurons (Krishnan et al., 2015).

In the PFC, PNN are mostly associated with PV^+^ neurons, with minor expression in some deeper cortical layer excitatory neurons (Mauney et al., 2013; Ueno et al., 2017). Expression of PNN is absent in infancy and increases throughout postnatal development in PFC (Ueno et al., 2017). It is well known that the PNN level is heterogeneously distributed with a wide range of PV^+^ neurons (Cisneros-Franco et al., 2018; Sigal et al., 2019), although how PV intensity is accurately regulated is poorly understood. A recent analysis of PV levels in neurons under various physiological and pathologically relevant conditions [such as memory formation (Donato et al., 2015) and in schizophrenia (Mukherjee et al., 2019)] have revealed a critical contribution of dynamic regulation of PV levels. The quantitative relationship between PV and PNN in a given neuron is poorly understood in both health and diseases. To date, only one study reported a positive correlation between PV and PNN within the same neuron in mice (Slaker et al., 2018). But whether or how this relationship may be regulated or altered during development or in ASD mice is currently unknown. Given the importance of these two molecules in plasticity and diseases, this analysis is important and may provide new insight into the pathogenesis of ASD.

In this study, we performed quantitative analysis on the intensities of PV and PNN in control (saline) and VPA mice, and the relationship between PV and PNN in a single PV^+^ neuron in the prelimbic PFC (PrL-PFC). We found the relationship between PV and PNN intensities is altered in VPA mice, likely due to an “abnormal” subpopulation of neurons with an altered PV-PNN relationship. Enzymatic digestion of PNN corrects the PV expression in the adult VPA mice, thus may remove this subpopulation and restore the normal PV-PNN relationship.

## Materials and Methods

### Animals

ICR mice were housed and fed at Peking University Shenzhen Graduate School as our previous study did (Gao et al., 2019). All experiments have been approved by the Peking University Shenzhen Graduate School Animal Care and Use Committee and were performed following the ARRIVE guidelines on the Care and Use of Experimental Animals. Adult mice were allowed to mate overnight, and the day of vaginal plug detection was defined as embryonic day 0.5 (E0.5). At E12.5, 500 mg/kg valproic acid (VPA; Sigma–Aldrich, Catalog #P4543) dissolved in 0.9% saline at a concentration of 100 mg/ml or the matched amount of saline were intraperitoneally injected, respectively. Females were housed individually and allowed to raise their litters. The offspring were weaned on postnatal day 21 (P21). All subsequent experiments were performed only on the male offspring. Male mice from VPA-injected and saline-injected were perfused for immunostaining at juvenile (P22), adolescence (P35–P36), and adult (P60–P80). For perfusion, the animals were deeply anesthetized before perfusion and transcardiac perfusion with PBS was followed by perfusion with 4% paraformaldehyde in chilled PBS.

### Enzymatic Degradation of PNN

To disrupt PNN in PrL-PFC, adult mice underwent a stereotaxic injection of the bacterial enzyme chondroitinase ABC (ChABC; Sigma–Aldrich, Catalog #C3667) or vehicle as control [Buffer containing 50 mM Tris, 60 mM sodium acetate and 0.02% bovine serum albumin (pH = 8.0)]. ChABC was prepared to a final concentration of 40 U/ml (Faini et al., 2018). Mice were anesthetized with (2–2.5% isoflurane) during the injection. The location for the injection site is AP+1.98, ML ± 0.3, and DV-2.2 (in mm, relative to Bregma). Injection volume was 250 nl and injection rate of 80 nl/min. After injection, the opening was closed using tissue adhesive (Vetbond, Catalog #1469SB) and mice were returned to the home cage for recovery. Three days after injection, mice were perfused as previously described for immunostaining.

### Brain Tissue Processing and Immunostaining

Brain tissue processing was performed based on previously published methods (Donato et al., 2015; Gao et al., 2019). In brief, upon removal, whole brains were fixed overnight in 4% paraformaldehyde at 4°C, followed by cryoprotection with 30% sucrose for 48 h. Brains were then paraffin-embedded and sectioned at 40 μm on a cryostat (Leica CM1860 UV, Germany). PrL-PFC sections were selected and double-stained with biotin-conjugated lectin from *Wisteria floribunda* (biotinylated WFA; Sigma, Catalog #L1516) and anti-parvalbumin antibody (anti-PV; Abcam, Catalog #ab11427) in a single reaction to control for potential variation in staining and tissue processing. Sections were washed three times with PBS, blocked with 10% normal goat serum for 1 h at room temperature, permeabilized with PBS containing 0.4% Triton X-100 at the same time, and then incubated with anti-PV (1:2,000) and biotinylated WFA (1:1,000) overnight at 4°C. Following subsequent washes in PBS with 0.4% Triton X-100 and incubated with Alexa Fluor^®^ 488 goat anti-rabbit IgG (1:500, Life Technologies, Catalog #A11008) and Streptavidin, Alexa Fluor^TM^ 546 conjugate (1:500, Invitrogen, #S11225) for 1 h at room temperature, sections were then washed three times in PBS with 0.4% Triton X-100. Three to four location-matched sections were selected from each mouse, and five mice were included in each group for each stage (total of 15–20 images/group). To avoid the batch effect, all samples were stained together.

### Analysis of PV and PNN Intensities

All brain sections were imaged on a confocal microscope (Zeiss-LSM700, Germany) using 20× objective (NA 0.75). Laser intensity, gain, offset, and pinhole settings were kept the same for all imaging sessions. During image acquisition, we first identified the PrL-PFC region using landmarks under a 10× objective, placed it in the center, and switched to the 20× objective. Since there are no PV^+^ neurons in layer 1, we started from layer 2. We used the entire image taken under the 20× objective as PrL-PFC, including layers 2–6. Images were taken in a z-stack (10 μm) within the center of the tissue section, containing 10 focal planes (1 μm/plane) in the PrL-PFC. Quantification of PV and WFA-labeled PNN numbers were counted from a total of 15–20 images for each group. The number of cells was semi-automatically counted and the experimenters were blinded to the experimentation or manipulations. For PNN and PV intensity, a semi-automated analysis, “PIPSQUEAK” macro plugin^1^, was used described in Slaker et al. (2016). The macro runs in “semi-automatic mode” on a selected region of interests (ROIs) to identify individual PV and PNN. ROIs were identified around PNN or PV and the mean intensity for each neuron was measured. Intensities of PV and PNN associated with each neuron were recorded, and only neurons with co-localized PV and PNN were analyzed and reported.

### Statistical Analysis

#### Comparisons of Average Levels of Various Variables

We calculated the means of variables, including densities of PV^+^ neurons and PNN, percentage of PV^+^ neurons ensheathed by PNN, and of PNN surrounding PV^+^ neurons for each mouse. Linear regression models were applied to compare the means of these variables between saline and VPA groups. Specifically, models with categorical variables of age group (i.e., P22, P35, and adult) and treatment (i.e., VPA and saline) were first fitted. Subsequently, we constructed models that included additional interaction-effect terms of age group and treatment and compared the models with and without the interaction-effect terms using likelihood ratio tests (LRTs). If an interaction effect was not statistically significant for an outcome, then we used the model without the interaction-effect terms for the analysis and made an overall comparison of the outcome between saline and VPA groups for all age groups. Otherwise, we compared the outcome between saline and VPA groups separately for three age groups using the model with interaction-effect terms and further applied the false discovery rate (FDR) to adjust *P* values for multiple comparisons.

#### Analysis of Paired Data of PV and PNN Intensities

Differences in PV and PNN intensities between saline and VPA groups were further analyzed using the paired data of PV and PNN intensities (i.e., from the same neuron). Kolmogorov–Smirnov tests were employed to assess disparities in the distribution of PV and PNN intensities between saline and VPA groups and between different age groups. Geometric means were calculated for PV and PNN intensities since the data are skewed to the right. Linear mixed models were applied to compare the logarithm transformations of PV intensity [log(PV)] and PNN intensity [log(PNN)] between saline and VPA groups, and between different age groups, accounting for the clustering effects of mouse and brain section within a mouse. Also, we explored correlations between PV and PNN intensities, elucidating whether the correlations differ in saline and VPA groups for different age groups using a linear mixed model, with log(PV) as the dependent variable and categorical variables of age group and treatment, log(PNN), an interaction-effect term of log(PNN) and age group and an interaction-effect term of log(PNN) and treatment as independent variables. Specifically, the coefficient of log(PNN), i.e., the slope of the fitted line of the regression model, was applied to indicate the association between log(PV) and log(PNN). Preliminary analysis suggested that a subpopulation of low PV and high PNN may influence the correlation between PV and PNN intensities. To determine whether the subpopulation has such impact, we further fitted models to explore the associations between log(PV) and log(PNN) for two subpopulations: the subpopulation with altered PV-PNN relationship (“abnormal” subpopulation) and others (“normal” subpopulation). We hypothesized that the “abnormal” subpopulation was those with PV intensity ≤ a threshold of PV intensity (thrsPV) and PNN intensity ≥ a threshold of PNN intensity (thrsPNN). We used four selection strategies to choose the combinations of thresholds of PV and PNN intensities for adult mice.

### Selection Strategy 1

In general, we selected combinations of thresholds of PV and PNN intensities to meet the following criteria: (1) higher median proportion of “abnormal” subpopulation for VPA than for saline adult mice; (2) the stronger association between log(PV) and log(PNN) in “normal” subpopulation than in “abnormal” subpopulation (i.e., the larger estimated slope of fitted line in the regression model for “normal” subpopulation than for “abnormal” subpopulation); (3) no difference in the association of log(PV) with log(PNN) between saline adult mice and “normal” subpopulation of VPA adult mice (i.e., statistically non-significant difference in the slopes of fitted lines in the regression models between saline adult mice and “normal” subpopulation of VPA adult mice); (4) different associations of log(PV) with log(PNN) between saline adult mice and “abnormal” subpopulation of VPA adult mice (i.e., statistically significant difference in the slopes of fitted lines in the regression models between saline adult mice and “abnormal” subpopulation of VPA adult mice); (5) no association between log(PV) and log(PNN) in “abnormal” subpopulation of VPA adult mice (i.e., statistically non-significant difference between the slope of fitted lines in the regression models for “abnormal” subpopulation of VPA adult mice and zero). Based on these, we subsequently calculated an indicator for comparing the combinations of thresholds as:

*area* = [thres_PV_ − min(PV)] × [max(PNN) − thres_PNN_]/2

where min(PV) and max(PNN) are the minimum PV intensity and maximum PNN intensity in the “abnormal” subpopulation in the VPA adult mice.

### Selection Strategy 2

We chose the “abnormal” subpopulation by relaxing the criterion (5) to allow the slope of fitted lines for “abnormal” adult VPA mice to be smaller than zero.

### Selection Strategy 3

We selected the combinations of thresholds that corresponded to the smallest *area* instead of the largest *area*.

### Selection Strategy 4

We chose the “abnormal” subpopulation by relaxing the criterion (5) to allow the slope of fitted lines for “abnormal” subpopulation of adult VPA mice to be smaller than zero and select the combinations of thresholds that corresponded to the smallest *area*.

Fractions of “abnormal” subpopulation in adult mice of VPA and saline groups were compared using the Mann-Whitney U test. A similar analytical strategy was used to compare the distribution of PV intensity and average levels of log(PV) between vehicle and ChABC groups.

In this study, “N” represents the number of statistical units (mice or cells). Since, we aim to determine how individual cells would change under these different contexts/manipulations, we treat each cell as a unit when comparing average expression level and distribution of PV and PNN intensities. The assessment of the correlation between PV and PNN is also based on single cells as the statistical units. In other cases, mice were treated as the statistical units. A significance level of 0.15 was applied for testing the interaction effects. *P*-value <0.05 was considered statistically significant for testing the differences in outcomes between saline and VPA groups and between vehicle and ChABC groups. All analyses were performed using R software version 3.6.2 (R Foundation for Statistical Computing) and Graphpad Prism 6.0.

## Results

### Altered Density of PV^+^ and PNN^+^ Neurons in Prelimbic PFC of VPA Mice Across Development

**Figure 1:**
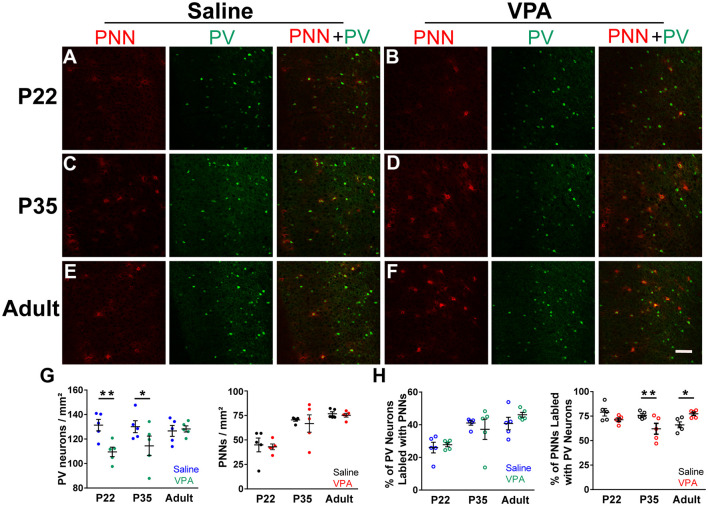
Density of parvalbumin (PV)^+^ neurons and *Wisteria floribunda* agglutinin (WFA)^(+)^ perineuronal nets (PNN) in prelimbic prefrontal cortex (PrL-PFC) across development in saline and valproic acid (VPA) mice. **(A–F)** Representative images of PV (green) and PNN (red) staining in the PrL-PFC of saline and VPA mice. Scale bar, 100 μm. **(G)** The density of PV^+^ neurons (left) and PNN (right) in PrL-PFC. **(H)** Percentage of PV^+^ neurons ensheathed by PNN (left) and PNN wrapped PV^+^ neurons (right). Blue, green, black, and red points in panel **(G)** represent the density of PV^+^ neurons in the saline group and VPA group, the density of PNN in the saline group and VPA group, respectively. Blue, green, black, and red circles in panel **(H)** represent the percentage of PV^+^ neurons ensheathed by PNN in the saline group and VPA group, PNN wrapped PV^+^ neurons in the saline group and VPA group, respectively. Black lines indicate Mean ± SEM. *n* = 5 mice for each group. Linear regression models were applied to compared various outcomes between groups using mice as the statistical units. *P < 0.05, **P < 0.01.

To understand changes in PV and PNN levels in the VPA model of autism, we first examined the densities of PV^+^ neurons and PNN in PrL-PFC in saline and VPA mice. Representative immunofluorescence images from PrL-PFC of mice at P22, P35, and adult (P60–80) are shown in Figures 1A–F. Interaction effects between age group and treatment were statistically significant for the density of PV^+^ neurons (*P* = 0.052; the significance level is set to 0.15 for interaction effects) and percentage of PNN surrounding PV^+^ neurons (*P* < 0.001; Supplementary Table 1). A lower PV^+^ neuron density was found in VPA group compared to saline group at P22 (mean difference = −21.85; *P* = 0.002) and P35 (mean difference = −15.67; *P* = 0.027), but not in adult (*P* = 0.818) (Supplementary Table 2 and Figure 1G). The percentage of PV^(+)^+PNN^(+)^ in all PNN^+^ neurons was lower in VPA group than in saline group at P35 (mean difference = −13.76; *P* = 0.003), but higher in VPA group in adult (mean difference = 11.32; *P* = 0.014; Supplementary Table 2 and Figure 1H). Thus, we found age-dependent changes in the density of PV^+^ and/or PNN^+^ neurons in the VPA mice, providing the basis for a more detailed analysis on changes in PV and PNN intensities in VPA mice.

### Higher PNN but Lower PV Intensity in PFC Neurons in VPA Mice

**Figure 2:**
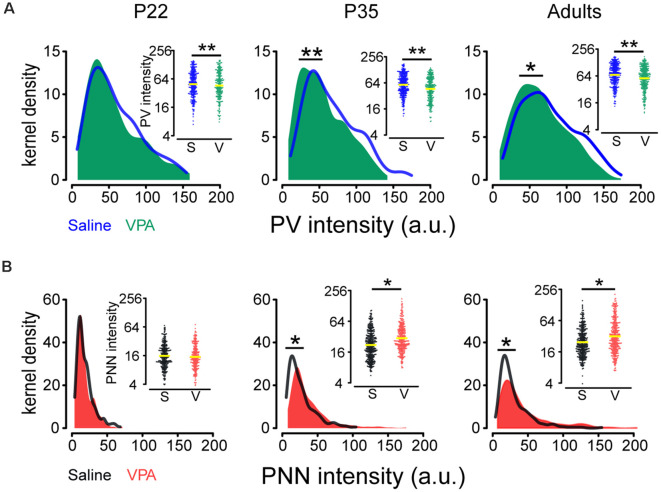
Comparisons of PV and PNN intensities in the PrL-PFC between saline and VPA mice. **(A)** Kernel density estimation of PV intensity in saline and VPA mice for different ages. Insert: Beeswarm plots of PV intensity. **(B)** Kernel density estimation of PNN intensity in saline and VPA mice for different ages. Insert: Beeswarm plots of PNN intensity. Yellow lines in inserts indicate geometric means of PV and PNN intensities. Kolmogorov–Smirnov tests were employed to assess disparities in the distribution of PV and PNN intensities between saline and VPA groups using cells as the statistical units. Linear mixed models were applied to compare the means of logarithm transformations of PV intensity [log(PV)] and PNN intensity [log(PNN)] between groups using cells as the statistical units. *N* = 287, 366, and 351 cells in P22, P35, and adult saline groups, respectively. *N* = 242, 305, and 348 cells in P22, P35, and adult VPA groups, respectively. *P < 0.05, **P < 0.01.

Analysis of PV and PNN intensities on the same neuron requires a quantitative measurement of both PV and PNN intensities. Thus, we followed the methods of Slaker et al. (2016; see “Materials and Methods” section) and compared changes (average expression level and distribution) in PV and PNN intensities in saline and VPA mice. Interaction effects between age group and treatment were statistically significant for log(PNN) (*P* = 0.049; Supplementary Table 1) while not for log(PV). log(PV) was on average lower in the VPA group than in saline group [mean difference in log(PV): −0.15; ratio of PV intensity (VPA/saline) = 0.86; *P* = 0.002; Figure 2A], while PNN intensity was on average higher in the VPA group than in saline group for P35 [mean difference in log(PNN): 0.28; ratio of PNN intensity = 1.33; *P* = 0.027] and adult [mean difference in log(PNN): 0.26; ratio of PNN intensity = 1.30; *P* = 0.040; Figure 2B, Supplementary Table 2].

Since, PV and PNN intensities varied widely and PV^+^ neurons are be likely composed of different subpopulations according to their intensity values (Donato et al., 2015), we employed Kolmogorov-Smirnov tests to assess disparities in their distributions between saline and VPA groups. We found a leftward shift in PV intensity distribution (P35: *P =* 0.003; adult: *P* = 0.013; Figure 2A) and a rightward shift in PNN intensity distribution (P35: *P* < 0.001; adult: *P* < 0.001; Figure 2B) in VPA mice, compared to saline group at P35 and adult.

### Correlations Between PV and PNN Differed Between Saline and VPA Groups

**Figure 3:**
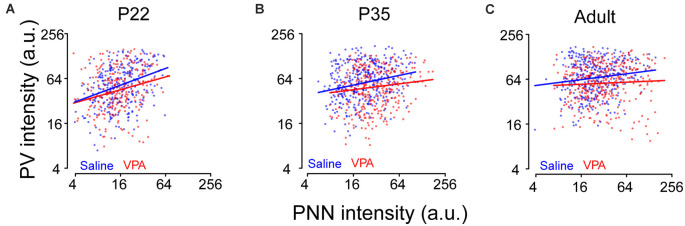
Relationship between PV and PNN intensities for a given neuron at different ages in saline and VPA mice. Logarithmic scales were used to present the values of PV and PNN intensities. Lines represent the relationship between the logarithm transformations of PV intensity [log(PV)] and PNN intensity [log(PNN)]: log(PV) = a×log(PNN) + b. **(A)** At P22, *a* = 0.37, *b* = 2.92 in saline group; *a* = 0.29, *b* = 3.02 in VPA group. **(B)** At P35, *a* = 0.22, *b* = 3.36 in saline group; *a* = 0.13, *b* = 3.45 in VPA group. **(C)** In adult, *a* = 0.13, *b* = 3.78 in saline group; *a* = 0.05, *b* = 3.88 in VPA group. Linear mixed models were applied to explore the correlation between log(PV) and log(PNN) using cells as the statistical units. *N* = 287, 366, and 351 cells in P22, P35, and adult saline groups, respectively. *N* = 242, 305, and 348 cells in P22, P35, and adult VPA groups, respectively.

To further understand the relationship between PV and PNN levels in the same neuron and whether a different relationship may emerge in the VPA mice, we examined this relationship in saline and VPA groups at different ages. As expected, the correlation between log(PV) and log(PNN) differed between saline and VPA groups (*P =* 0.034) at all ages, with a stronger positive association in a saline group than in the VPA group (Figures 3A–C). Also, it seemed that a distinct subpopulation appeared who lied in the right bottom area in VPA adult mice when comparing the data distribution of PV-PNN with saline adults (slope = 0.13 and 0.05 in saline and VPA group, respectively; Figure 3C).

### Analysis of a Potential Subpopulation in VPA Adult Mice

**Figure 4:**
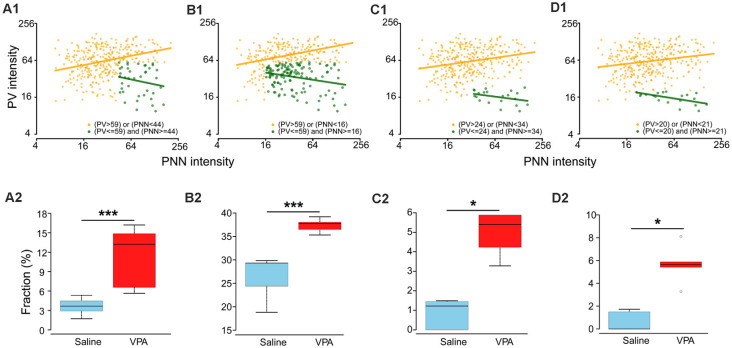
Different assumptions of a distinct subpopulation in VPA adults. **(A1,B1,C1,D1)** Relationships between PV and PNN intensities for two subpopulations in adult VPA mice. Dark green and orange dots are “abnormal” and “normal” subpopulations. Dark green and orange lines represent the relationship between the logarithm transformations of PV intensity [log(PV)] and PNN intensity [log(PNN)]: log(PV) = a×log(PNN) + b. For **(A1)**, “normal” subpopulation: *a* = 0.25, *b* = 0.04; “abnormal” subpopulation: *a* = −0.27; *b* = 0.21. For **(B1)**, “normal” subpopulation: *a* = 0.24, *b* = 0.04; “abnormal” subpopulation: *a* = −0.19; *b* = 0.06. For **(C1)**, “normal” subpopulation: *a* = 0.18, *b* = 0.04; “abnormal” subpopulation: *a* = −0.17; *b* = 0.10. For **(D1)**, “normal” subpopulation: *a* = 0.15, *b* = 0.04; “abnormal” subpopulation: *a* = −0.20; *b* = 0.07. **(A2,B2,C2,D2)** Boxplots of fractions of “abnormal” subpopulation in adult mice for saline and VPA groups. Thick lines in boxes indicate medians of fractions. The lower and upper bounds of boxes represent the 1st (Q1) and 3rd quartiles (Q3) of fractions. IQR = Q3 − Q1. Thin lines located outside boxes are the minimum fractions and the smaller of the maximum fractions and Q3+1.5 × IQR. Circles were used to indicate values outside the ranges between Q1–1.5 × IQR and Q3 + 1.5 × IQR. Fractions of “abnormal” subpopulation in adult mice of VPA and saline groups were compared using the Mann–Whitney *U* test. *P < 0.05, ***P < 0.001.

There are at least two possible causes for the observed PV-PNN relationship in the VPA mice: (1) a subpopulation either emerging from an existing one or as a new one with distinct PV/PNN values as a result of VPA treatment; (2) a shift of the entire cell population for the emerging of the new subpopulation. The second cause cannot be confirmed due to technical reasons. We thus focused on the first possibility. Based on the criteria described in the “Materials and Methods” section, we obtained four results on the “abnormal” subpopulation in the VPA mice which were corresponded to selection strategies 1–4. Results 1 and 2 were based on selecting a combination of thresholds corresponding to the largest *area*, which also provided the largest number of “abnormal” cells. Result 1: we obtained the “abnormal” subpopulation (PV ≤ 59 a.u. and PNN ≥ 44 a.u.) in which the association between log(PV) and log(PNN) was not statistically significant (slope = −0.27, *P* = 0.206; Figure 4A1, dark green dots), while a positive correlation was observed in the remaining subpopulation (i.e., “normal” subpopulation; slope = 0.25, *P* < 0.001; Figure 4A1, orange dots). In addition, the difference in the proportion of “abnormal” subpopulation in VPA (median: 13.2%) was statistically larger than in saline groups (median: 3.7%; *P* = 0.008; Figure 4A2). We may consider this result as an “abnormal” subpopulation emerging with VPA treatment to be a new subpopulation. Result 2: the “abnormal” subpopulation (PV ≤ 59 a.u. and PNN ≥ 16 a.u.) had a negative association between log(PV) and log(PNN) (slope = −0.19, *P* = 0.002; Figure 4B1; dark green dots), and a positive correlation in the remaining subpopulation (slope = 0.24, *P* < 0.001; Figure 4B1, orange dots). Both the proportion of “abnormal” subpopulation in VPA (median: 29.3%; Figure 4B2) and saline group (median: 37.7%; Figure 4B2) accounted for about 30%, even though significant differences exist between them. This result supports the possibility of the subpopulation in VPA mice emerging from an existing subpopulation in the saline mice.

We then selected the combinations of thresholds corresponding to the smallest *area*. Result 3: the “abnormal” subpopulation was those with PV ≤ 24 a.u. and PNN ≥ 34 a.u. (slope = −0.17, *P* = 0.133; Figure 4C1, dark green dots), and with a positive correlation in the “normal” subpopulation (slope = 0.18, *P* < 0.001; Figure 4C1, orange dots). But the medians of proportions of “abnormal” subpopulation for VPA and saline adult groups were 5.4% and 1.2%, respectively (Figure 4C2). Result 4: the “abnormal” subpopulation had PV ≤ 20 a.u. and PNN ≥ 21 a.u. (slope = −0.20, *P* = 0.014; Figure 4D1, dark green dots), with a positive correlation in the remaining subpopulation (slope = 0.15, *P* < 0.001; Figure 4D1, orange dots). The medians of proportions of “abnormal” subpopulation for VPA and saline adult groups were 5.6% and 0.0%, respectively (Figure 4D2). We think that such low proportions of “abnormal” subpopulations obtained in Results 3 and 4 may be too small to drive or underlie the pathology in VPA mice.

### Removing PNN Restores the PV Expression in Adult VPA Mice

**Figure 5:**
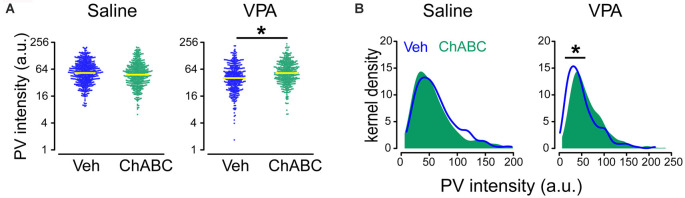
Restored PV expression in ChABC-treated VPA adult mice. **(A)** Beeswarm plots of PV intensity from all neurons analyzed in saline and VPA mice treated by Veh and ChABC, respectively. **(B)** Kernel density estimation of PV intensity for all neurons analyzed in saline and VPA mice treated by Veh and ChABC, respectively. Yellow lines in panel **(A)** indicate geometric means of PV intensity. Linear mixed models were applied to compare the means of logarithm transformations of PV intensity [log(PV)] between groups using cells as the statistical units. Kolmogorov–Smirnov tests were employed to assess disparities in the distribution of PV intensity between groups using cells as the statistical units. *N* = 512, 448, 381, and 411 cells in Saline-vehicle, Saline-chABC, VPA-vehicle, and VPA-chABC groups, respectively. *P < 0.05.

It is generally viewed that a higher PNN level reduces/limits neural plasticity (Pizzorusso et al., 2002; Sigal et al., 2019), and hence the presence of this subpopulation of high-PNN/low-PV group in the VPA mice may be caused by abnormally high PNN level. If this hypothesis is correct, reducing the PNN level should correct this abnormality in the PV level. To test this hypothesis, we injected ChABC into PrL-PFC of adult VPA mice (P80) and analyzed changes (average expression level and distribution) in PV intensity in PrL-PFC. Sample images of ChABC treatment in both saline and VPA mice were shown in Supplementary Figure 1, and PNNs were degraded in both saline and VPA group (Supplementary Figure 1, red). Interaction effects between treatment (VPA) and ChABC on log(PV) were statistically significant (*P =* 0.009; Supplementary Table 1; Figure 5A). Intensity level of PV was on average higher in the ChABC-treated VPA group [mean difference in log(PV) = 0.27; ratio of PV *intensity* (ChABC/vehicle) = 1.31; *P =* 0.039; Figure 5A; Supplementary Table 2]. No statistically significant difference in log(PV) was found between vehicle and ChABC groups of saline mice (*P* = 0.348; Figure 5A; Supplementary Table 2). Also, in VPA mice, the ChABC group had a different PV intensity distribution (rightward shift), compared to the vehicle group (*P* < 0.001), but this difference was absent in saline mice (*P* = 0.104; Figure 5B). Moreover, PV expression level (including both average expression level and distribution) was almost identical to those in vehicle-treated saline mice (average expression level: *P* = 0.958; distribution: *P* = 0.872). These results collectively suggest that a high PNN level may influence PV level. Enzymatic digestion of PNN restores the PV expression, thus may remove the possible “abnormal” subpopulation and restore the normal PV-PNN relationship in the adult VPA mice.

### Developmental Changes in PV and PNN Intensities in Saline and VPA Mice

**Figure 6:**
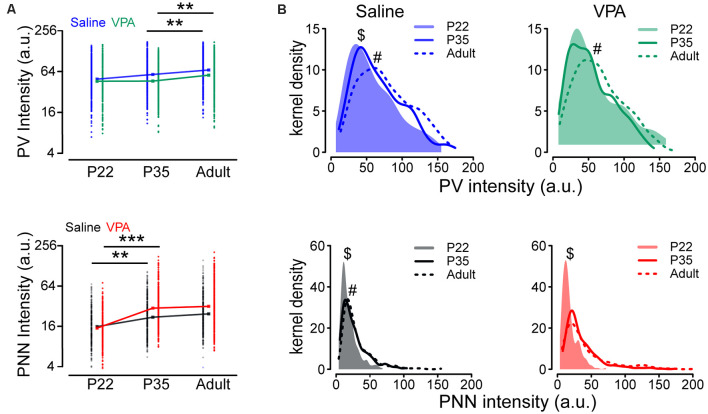
Altered intensities of PV and PNN in VPA mice during postnatal development. **(A)** PV and PNN intensities in PrL-PFC of P22, P35, and adult saline and VPA mice. **(B)** Kernel density estimation of the PV and PNN intensities in PrL-PFC of P22, P35, and adult mice. Points in panel **(A)** represent observed PV and PNN intensities, while rectangles indicate geometric means. Linear mixed models were applied to compare the means of logarithm transformations of PV intensity [log(PV)] and PNN intensity [log(PNN)] between groups using cells as the statistical units. Kolmogorov-Smirnov tests were employed to assess disparities in the distribution of PV intensity between groups using cells as the statistical units. *N* = 287, 366, and 351 cells in P22, P35, and adult saline groups, respectively. *N* = 242, 305, and 348 cells in P22, P35, and adult VPA groups, respectively. **P < 0.01, ***P < 0.001. ^$^statistical significance between P22 and P25; ^#^significance between P35 and adult. *P*-values in Supplementary Table 3.

We addressed whether the development of PV and PNN (average expression level and distribution) is altered during development in VPA mice. The difference in the mean log(PV) between P22 and P35 was not statistically significant (*P* = 0.111) but the level of an adult was on average higher than that at P35 (*P* = 0.008), in both saline and VPA groups (Figure 6A and Supplementary Table 3). Unlike log(PV), we found that the mean log(PNN) was higher at P35 than that at P22, in both saline (*P* = 0.009) and VPA group (*P* < 0.001), but the difference between P35 and adult was statistically non-significant (saline: *P* = 0.396; VPA: *P* = 0.507), suggesting that the developmental increase in PNN intensity occurs at early ages (Figure 6A and Supplementary Table 3). We then compared distributions of PV and PNN during development between saline and VPA groups. Different from results of average level of expression, PV distributions showed a continuous rightward shift from P22 to P35 (*P* = 0.014) and from P35 to adult (*P* = 0.003) in saline group, as well as PNN distributions (P22 vs. P35: *P* < 0.001; P35 vs. adult: *P* = 0.045; Supplementary Table 4 and Figure 6B). However, for VPA mice, the difference in PV distribution between P22 and P35 was not statistically significant (*P* = 0.642; Supplementary Table 4 and Figure 6B), and the disparity in PNN distribution was statistically non-significant between P35 and adult (*P* = 0.235; Supplementary Table 4 and Figure 6B).

In summary, changes in the average expression levels of PV and PNN did not always match with their distributions, in both saline and VPA groups. The main differences in PV and PNN intensity between saline and VPA during development are in their distributions rather than their average levels. This finding is consistent with our observation of the emergence of a PV subpopulation in the VPA mice.

## Discussion

We have focused on quantitative changes in the PV and PNN intensities and the relationship between them in the same neuron in a VPA model of ASD. Our key finding is an altered relationship between PV and PNN levels, and the emergence of an abnormal subpopulation with an unbalanced PV-PNN relationship, in the PrL-PFC of VPA mice. Reducing PNN level with ChABC treatment *in vivo* restores PV expression and may have eliminated this distinct subpopulation from VPA mice, suggesting that PNN level may determine/control PV level. We suggest that altered interaction between PNN and PV may contribute to the pathophysiological changes underlying ASD.

Although many prior studies have examined the density of PV^+^ neurons, PNN under both physiological and disease conditions, quantitative analysis of the intensities of PV and PNN within the same neuron is limited, and comparison between these two parameters in the same neurons is almost not existing until Slaker et al. (2018) demonstrated a positive correlation between PV and PNN (WFA^+^) intensity within the same neuron using linear regression analysis. However, the functional significance of this correlation and whether the relationship may be altered in psychiatric diseases is poorly understood. To our knowledge, this is the first report of an abnormal relationship between PV and PNN levels in a given neuron in ASD model animals. We explored correlations between PV and PNN using linear mixed models which accounted for the clustering effects of mouse and brain section. Unlike the units sampled at the mouse level in the study, observations at the cellular level are not independent of each other, as cells in a given brain slice and mouse are more likely to behave/function similarly compared to those from different animals. Each brain section within a mouse and each mouse are thus treated as clusters in this study. It is inappropriate to analyze such data using simple linear regression models which require that observations be independent of each other. Thus, linear mixed models were applied to compare the logarithm transformations of PV intensity [log(PV)] and PNN intensity [log(PNN)], accounting for the clustering effects stated above. In saline mice, there is a positive relationship between PV and PNN levels across developmental ages and in adulthood, suggesting that maintaining this positive correlation is likely critical for the proper functioning of PV^+^ neurons. This suggestion is consistent with the observations that PV is known to regulate electrophysiological properties of PV^+^ neurons (such as spike frequency and spike frequency adaption; Orduz et al., 2013) while PNN in intrinsic excitability and synaptic transmission, and hence their levels proportional to each other may be suited or even required for balanced action in these areas. In that regard, low PNN-low PV^+^ neurons may represent a subpopulation of higher plasticity, while high PNN-high PV presents a subpopulation of low plasticity. PV intensity, as well as increased PNN intensity around PV+ neurons (but not in the same neurons), was also found in the ventral hippocampus in an excessive anxiety mice model using repeated maternal separation with early weaning (MSEW), and altered PV and PNN may be related to MSEW-induced anxiety and hyperactivity (Murthy et al., 2019). However, the relationship between PV and PNN intensities was not explored in their study. We suppose that a subpopulation is abnormal in PV and PNN relationship and thus the functions carried out by this subpopulation may be altered in the VPA mice. The impact of this subpopulation on normal functions and ASD pathology needs to be examined in future studies.

Based on the experimental PNN and PV data, we have tested various potential models as to how the PV-PNN relationship may evolve as a result of VPA treatment to drive or underlie the pathology in VPA mice. To address this question, we did the following: (1) we considered the possibility of a shift in the entire population (PV and PNN values) as a result of VPA treatment to yield the distribution in the VPA adult mice. In other words, whether a mathematical transformation can be identified to relate the Saline population and VPA population. However, we were unable to come up with any solution due to technical reasons. (2) We have considered the possibility of a subpopulation emerging with VPA treatment, either from an existing subpopulation in VPA mice or as a new subpopulation. If a new subpopulation with distinct PV/PNN values emerged in VPA mice, we expect a substantially larger proportion in VPA than in saline mice, and our model A fits this model (Figure 4A). If an independent subpopulation exists in the saline/control mice which undergo changes by VPA to arrive at higher PNN levels (and corresponding PV levels), we expect a similar representation (measured as a percentage) in both saline and VPA brain, and our model B fits this model (Figure 4B). It will be quite interesting if we can identify this abnormal cell subpopulation experimentally. Distinct electrophysiological characteristics may exist for these “abnormal” neurons, but this requires measures of PV and PNN levels in live neurons which we are unable to do.

We found that ChABC restores PV expression and normalizes the distribution of PV intensities in adult VPA mice and hence it is likely that the abnormally high PNN level is likely the cause of low PV and the emerge of the abnormal subpopulation. This notion is consistent with a previous report on changes in PV levels lagged behind that of PNN after cocaine exposure (Slaker et al., 2018). What could cause this high PNN in the VPA mice? We noticed that neither genes encoding components of PNN nor PV were direct downstream targets of VPA (Fukuchi et al., 2009). Thus, these changes might be secondary. Notably, a key function of PNN is to protect their neurons against oxidative stress, while oxidative stress is one of the major proposed actions of VPA (Mabunga et al., 2015). Supporting this suggestion, increased oxidative stress was also demonstrated in young children treated with VPA (Verrotti et al., 2008), while antioxidants (such as vitamin E and ascorbic acid) given as a pretreatment or supplement have been demonstrated to attenuate VPA-induced fetal toxicity and malformations (Al Deeb et al., 2000; Zhang et al., 2010). Thus, elevated expression of PNN might be a protective response in these PV^+^ neurons against oxidative stress to ensure neuronal survival, albeit at the expanse of maldevelopment of other functions.

We found that PV intensity is not altered by ChABC treatment in the saline mice. There are a few potential reasons: (1) the duration of treatment. PV fluorescence and PV mRNA were decreased 7 days after the chABC injection in the mouse hippocampus (Yamada et al., 2015) and primary visual cortex (V1; Beurdeley et al., 2012). Notably, PV expression was not significantly altered by 3-day ChABC treatment in mouse V1 (Beurdeley et al., 2012). It is thus likely that longer ChABC or PNN removal may be required to trigger corresponding changes. (2) PV level modulation is not sensitive to or controlled by short-term alterations in the PNN level. In other words, physiological changes in PV level do not require changes in PNN level. (3) Changes in PNN level alone are not sufficient to induce changes in PV, other factors/triggers may be required. For example, these other factors may include processes that drive PV-related plasticity to occur. Faini et al. (2018) showed that the impact of PNN removal on synaptic transmission and visual processing is only revealed when coupled to sensory inputs. Furthermore, some studies have suggested that orthodenticle homeobox protein 2 (OTX2) needs to change first after PNN degradation which further leads to changes in PV level (Beurdeley et al., 2012). This scenario is consistent with somewhat minor and inconsistent reports on the impact of ChABC on PV^+^ neurons (such as spiking properties) in the absence of additional perturbations. For example, ChABC was found to reduce, increase or have no effect on the intrinsic excitability of PV^+^ neurons (Dityatev et al., 2007; Balmer, 2016; Chu et al., 2018; Hayani et al., 2018; Tewari et al., 2018).

We have limited our analysis to the PrL-PFC due to the significant contribution of it to ASD, but as a general question, it can be asked whether the above relationship between PV and PNN also holds for other brain regions, especially the sensory cortex where a high level of PNN is seen and the contribution of PNN and PV to development is more thoroughly examined. Since we were taking snap-shot at different ages, we were unable to examine longitudinal changes or evolvement of this newly identified subpopulation in VPA mice. Although alternatives exist, the simplest scenario is that the same group of PV^+^ neurons change gradually during development and contribute to our findings. Direct examination of this model is needed in future studies.

In summary, we showed that the relationship between PV and PNN is disrupted in VPA mice suggesting altered PV-PNN interactions may contribute to the pathogenesis and/or pathology of ASD.

## Data Availability Statement

The original contributions presented in the study are included in the article/Supplementary Material, further inquiries can be directed to the corresponding author/s.

## Ethics Statement

The animal study was reviewed and approved by Peking University Shenzhen Graduate School Animal Care and Use Committee.

## Author Contributions

QZ, BY, and DX conceived and designed the experiments. DX performed the experiments. LL did the statistical analysis. DX and LL finished the figures together. All authors contributed to the article and approved the submitted version.

## Conflict of Interest

The authors declare that the research was conducted in the absence of any commercial or financial relationships that could be construed as a potential conflict of interest.

## Footnotes

^1^http://labs.wsu.edu/sorg/research-resources/
