# Supplementary material for: Altered Relationship Between Parvalbumin and Perineuronal Nets in an Autism Model
Source: Front Mol Neurosci. 2021 Apr 12;14:597812. doi: 10.3389/fnmol.2021.597812 (PMC8072465; doi:10.3389/fnmol.2021.597812)
Supplement: Supplementary file 1 [file Data_Sheet_1.docx]

**Supplemental Materials**

**Figure S1.** **Representative images of PNN (red) and PV (green) staining in the prelimbic PFC after chABC treatment both in saline and VPA adult mice.**

Injection volume was 250 nL and injection rate of 80 nL/min. Three days after injection, mice were perfused with 4% PFA for immunostaining.

Bar=100 μm.

**Table S1.** Results of likelihood ratio tests for the interaction effects of age group and treatment on various dependent variables.

| Dependent variable | *P* |
| --- | --- |
| Density of PV^+^ neurons | **0.052** |
| Density of PNN | 0.973 |
| Percentage of PV^(+)^+PNN^(+)^ among PV^+^ neurons | 0.399 |
| Percentage of PV^(+)^+PNN^(+)^ among PNN^+^ neurons | **<0.001** |
| Logarithmic transformation of PV intensity | 0.378 |
| Logarithmic transformation of PNN intensity | **0.049** |
| Logarithmic transformation of PV intensity (ChABC) | **0.009** |

*Note. P*<0.15 was considered statistically significant for the tests for interaction effects and was in bold.

**Table S2.** Associations between treatment and various dependent variables.

| Dependent variable | Age group | Mean difference in dependent variable between VPA and saline groups (SEM) | *P*^#^ |
| --- | --- | --- | --- |
| Density of PV^+^ neurons | P22 | -21.85 (3.54) | **0.002** |
|  | P35 | -15.67 (3.54) | **0.027** |
|  | Adult | 1.63 (3.54) | 0.818 |
| Density of PNN | All | -2.40 (3.88) | 0.541 |
| Percentage of PV^(+)^+PNN^(+)^ among PV^+^ neurons | All | 1.08 (2.77) | 0.699 |
| Percentage of PV^(+)^+PNN^(+)^ among PNN^+^ neurons | P22 | -7.66 (2.30) | 0.096 |
|  | P35 | -13.76 (2.30) | **0.003** |
|  | Adult | 11.32 (2.30) | **0.014** |
| Logarithmic transformation of PV intensity | All | -0.15 (0.04) | **0.002** |
| Logarithmic transformation of PNN intensity | P22 | -0.07 (0.06) | 0.573 |
|  | P35 | 0.28 (0.06) | **0.027** |
|  | Adult | 0.26 (0.06) | **0.040** |
| Logarithmic transformation of PV intensity (ChABC) | Saline | -0.11 (0.05) | 0.348 |
|  | VPA | 0.27 (0.06) | **0.039** |

*Note. P* < 0.05 was in bold.

Abbreviation: SEM, standard error of the mean.

^#^ *P* values were adjusted using the false discovery rate for multiple comparisons.

**Table S3.** Associations between age group and dependent variables.

| Dependent variable | Category | Mean difference in dependent variable between age groups (SEM) | *P*^#^ |
| --- | --- | --- | --- |
| Logarithmic transformation of PV intensity | All |  |  |
|  | P35 vs P22 | 0.09 (0.06) | 0.111 |
|  | Adult vs P22 | 0.26 (0.06) | **<0.001** |
|  | Adult vs P35 | 0.16 (0.05) | **0.008** |
| Logarithmic transformation of PNN intensity | Saline |  |  |
|  | P35 vs P22 | 0.36 (0.12) | **0.009** |
|  | Adult vs P22 | 0.46 (0.12) | **0.002** |
|  | Adult vs P35 | 0.10 (0.12) | 0.396 |
|  | VPA |  |  |
|  | P35 vs P22 | 0.68 (0.12) | **<0.001** |
|  | Adult vs P22 | 0.76 (0.12) | **<0.001** |
|  | Adult vs P35 | 0.08 (0.12) | 0.507 |

*Note. P* < 0.05 was in bold.

Abbreviation: SEM, standard error of the mean.

^#^ *P* values were adjusted using the false discovery rate for multiple comparisons.**Table S4**. Kolmogorov-Smirnov tests for the comparisons for distributions of PV and PNN intensities between different age groups.

| Intensity | Group | *P* | |
| --- | --- | --- | --- |
|  |  | P22 *vs* P35 | P35 *vs* adult |
| PV | Saline | **0.014** | **0.003** |
| PV | VPA | 0.642 | **<0.001** |
| PNN | Saline | **<0.001** | **0.045** |
| PNN | VPA | **<0.001** | 0.235 |

*Note. P*<0.05 was in bold.
