# Supplementary figures and images for: Altered Relationship Between Parvalbumin and Perineuronal Nets in an Autism Model
Source: Front Mol Neurosci. 2021 Apr 12;14:597812. doi: 10.3389/fnmol.2021.597812 (PMC8072465; doi:10.3389/fnmol.2021.597812)

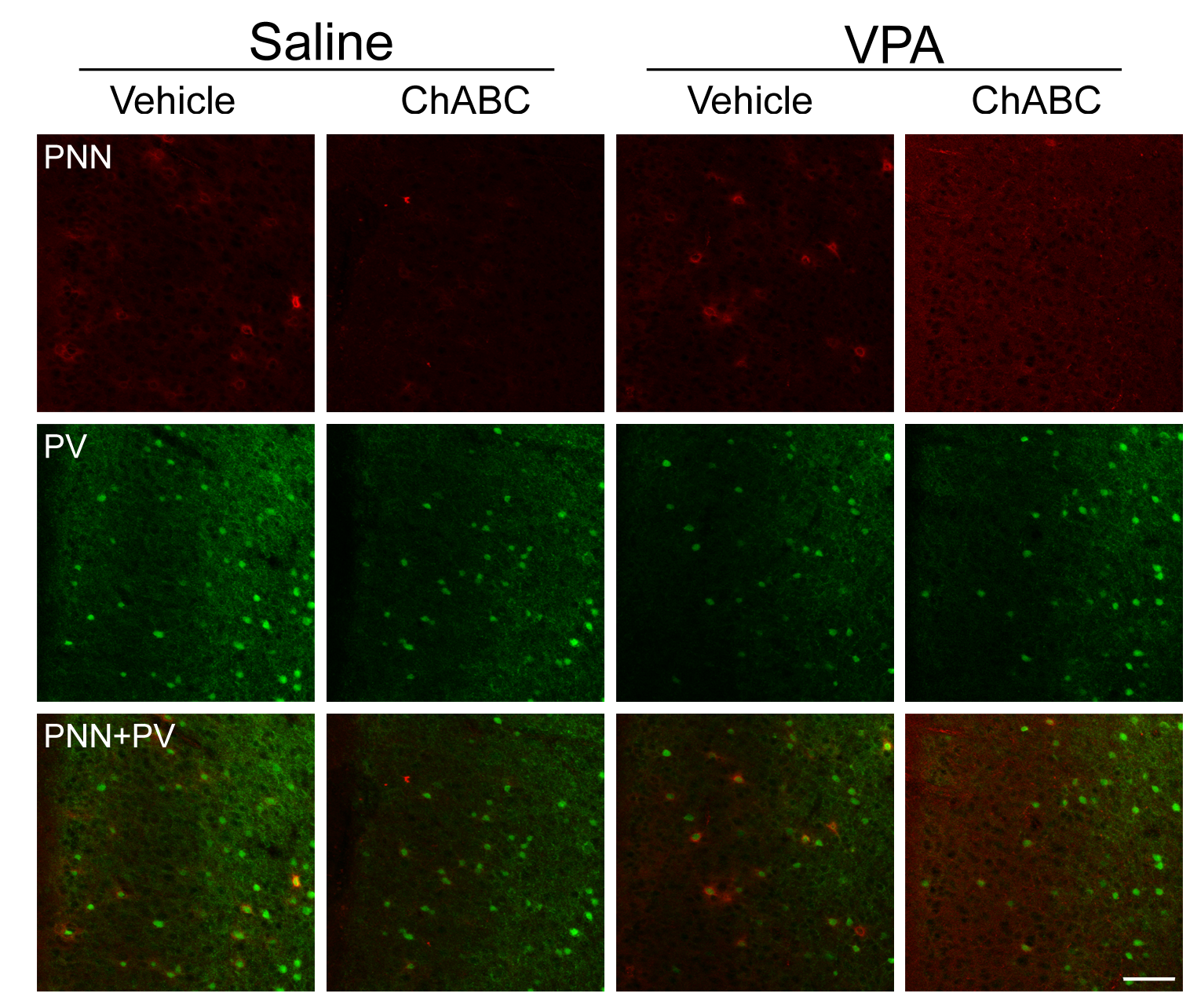

Supplement: Supplementary file 2 [file Image_1.tif]
